# Receipt of mastectomy and adjuvant radiotherapy following breast conserving surgery (BCS) in New Zealand women with BCS-eligible breast cancer, 2010–2015: an observational study focusing on ethnic differences

**DOI:** 10.1186/s12885-023-11248-9

**Published:** 2023-08-17

**Authors:** Karen Bartholomew, Mazin Ghafel, Sandar Tin Tin, Phyu S Aye, J Mark Elwood, Claire Hardie, Nina Scott, Jacquie Kidd, Reena Ramsaroop, Ian Campbell

**Affiliations:** 1 Te Whatu Ora Waitematā, Auckland, New Zealand; 2 Te Whatu Ora Te Toka Tumai Auckland, Auckland, New Zealand; 3 University of Auckland, 85 Park Road, Grafton, Auckland 1023 New Zealand; 4 Te Whatu Ora MidCentral, Palmerston North, New Zealand; 5 University of Auckland, Waikato Campus, Hamilton, New Zealand; 6 Auckland University of Technology, Auckland, New Zealand

**Keywords:** Breast cancer, Breast conserving surgery, Mastectomy, Adjuvant radiotherapy, Ethnicity, New Zealand

## Abstract

**Background:**

Women with early breast cancer who meet guideline-based criteria should be offered breast conserving surgery (BCS) with adjuvant radiotherapy as an alternative to mastectomy. New Zealand (NZ) has documented ethnic disparities in screening access and in breast cancer treatment pathways. This study aimed to determine whether, among BCS-eligible women, rates of receipt of mastectomy or radiotherapy differed by ethnicity and other factors.

**Methods:**

The study assessed management of women with early breast cancer (ductal carcinoma in situ [DCIS] and invasive stages I-IIIA) registered between 2010 and 2015, extracted from the recently consolidated New Zealand Breast Cancer Registry (now Te Rēhita Mate Ūtaetae NZBCF National Breast Cancer Register). Specific criteria were applied to determine women eligible for BCS. Uni- and multivariable analyses were undertaken to examine differences by demographic and clinicopathological factors with a primary focus on ethnicity (Māori, Pacific, Asian, and Other; the latter is defined as NZ European, Other European, and Middle Eastern Latin American and African).

**Results:**

Overall 22.2% of 5520 BCS-eligible women were treated with mastectomy, and 91.1% of 3807 women who undertook BCS received adjuvant radiotherapy (93.5% for invasive cancer, and 78.3% for DCIS). Asian ethnicity was associated with a higher mastectomy rate in the invasive cancer group (OR 2.18; 95%CI 1.72–2.75), compared to Other ethnicity, along with older age, symptomatic diagnosis, advanced stage, larger tumour, HER2-positive, and hormone receptor-negative groups. Pacific ethnicity was associated with a lower adjuvant radiotherapy rate, compared to Other ethnicity, in both invasive and DCIS groups, along with older age, symptomatic diagnosis, and lower grade tumour in the invasive group. Both mastectomy and adjuvant radiotherapy rates decreased over time. For those who did not receive radiotherapy, non-referral by a clinician was the most common documented reason (8%), followed by patient decline after being referred (5%).

**Conclusion:**

Rates of radiotherapy use are high by international standards. Further research is required to understand differences by ethnicity in both rates of mastectomy and lower rates of radiotherapy after BCS for Pacific women, and the reasons for non-referral by clinicians.

**Supplementary Information:**

The online version contains supplementary material available at 10.1186/s12885-023-11248-9.

## Background

Breast cancer is the most common cancer and the second leading cause of death for New Zealand women [1]. Breast cancer incidence and outcomes differ substantially by ethnicity. Māori and Pacific women had a higher incidence of breast cancer than New Zealand European women, but were less likely to be diagnosed via screening mammography, and therefore were frequently diagnosed at a more advanced stage [2–4]. Mortality from breast cancer in indigenous Māori and Pacific women is nearly double that of women of other ethnicities [5].

The standard treatment in New Zealand, according to the 2009 Guidelines for Early Breast Cancer Management [6], is the choice of breast conserving surgery (BCS) or mastectomy to all women who are eligible for BCS. BCS improves psychological (for women who wish to have breast conservation) and cosmetic outcomes [7] and when followed by adjuvant radiotherapy has demonstrated superior survival outcomes compared to mastectomy alone in observational studies [8–11]. Standard adjuvant radiotherapy usually involves a course of whole breast external-beam radiotherapy with or without a boost to the surgical tumour bed delivered daily over a number of weeks [6, 12]. Among women with node-negative disease, adjuvant radiotherapy reduces the 10-year risk of a first local recurrence of breast cancer by 15.4% and the 15-year risk of breast cancer-related mortality by 3.3% [13]. However, the duration of standard adjuvant radiotherapy may lead women to choose mastectomy rather than BCS [14].

Previous New Zealand research by colleagues using data from the Auckland and Waikato regional Breast Cancer Registers indicated that Māori and Pacific women with early breast cancer were significantly more likely to receive mastectomy [5, 15, 16], more likely to have treatment delays [5, 17], and less likely to receive radiotherapy after BCS than other women [5]. According to international studies, receipt of adjuvant radiotherapy for early stage breast cancer differed by socioeconomic status, ethnicity, age and travel distance for treatment [18, 19].

To better understand how ethnicity and other demographic and clinical factors influenced treatment choices in New Zealand women with breast cancer, this study used the data from the New Zealand Breast Cancer Register (NZBCR) and investigated (1) receipt of mastectomy in women with early breast cancer who were eligible for BCS, and (2) receipt of adjuvant radiotherapy in women who received BCS as their final surgery.

## Methods

### Data sources

This study is a population-based cross-sectional observational study, using the data of women with newly diagnosed early breast cancer, extracted from the New Zealand Breast Cancer Register (NZBCR) for the period of 2000–2015. The NZBCR is now a national nationwide database – from 2020. At the time of data extraction, the NZBCR had been prospectively consolidated data from four population-based opt-out registries in four areas: Auckland, Waikato, Wellington, and Christchurch. These registries represented data of nearly complete population-based series, accounted for approximately 67% of newly diagnosed breast cancers in New Zealand, with an opt-out rates of 1.6% in 2003–2012, and 0.1% in 2012–2020 [3]. The use of opt-out consent systems enhance reporting unbiased results [20].

### Study population

The study included all women with early breast cancer who were eligible for breast conserving surgery (BCS). Early breast cancer comprised of invasive breast cancer (stages I-IIIA) and ductal carcinoma in situ (DCIS). To be eligible for BCS, the following were excluded: unknown tumour size or a tumour size of ≥ 30 mm, no breast surgery, multifocal ipsilateral breast cancer, unknown invasive or in situ, stage IIIB-IV or unknown stage, lobular carcinoma in situ alone, being pregnant at the time of diagnosis, and male, transgender or unknown gender [21]. The study period was restricted to 2010–2015, for which the complete data was available across all four geographical regions.

### Variables

Ethnicity was the primary variable of interest, sourced from contributing hospital records and associated with the National Health Index (NHI) number. Guidance and requirements for the collection, recording and output of ethnicity data in New Zealand is covered in the Health and Disability Sector Ethnicity Data Standards [22]. In New Zealand ethnicity is self-identified using the standardised ethnicity question, and patients are able to identify multiple ethnic groups. For women who identified multiple ethnicities, the ethnicity used in this analysis was prioritised in the order Māori > Pacific > Asian > Other, allowing one assigned ethnicity per patient. The ‘Other ethnicity’ category is the largest and included women identifying as NZ European, Other European, and Middle Eastern Latin American and African (MELAA). In the source dataset only 12 (0.2%) of ethnicity data was missing. We acknowledge that there are known issues with ethnicity data quality using any routine dataset, including NHI ethnicity [23].

Demographic variables of interest included age at diagnosis, and measures of rurality and deprivation status. Rurality was categorised as rural or urban, according to Statistics NZ definitions based on patients’ residential address [24]. Deprivation status, which reflects individuals’ socioeconomic status, was calculated using NZ Deprivation (2013) decile scores [25], and categorised into quintiles 1–5 (1 = least deprived, 5 = most deprived).

Clinical variables included mode of diagnosis (whether detected by mammographic screening or symptoms), diagnosis year, type of healthcare facility (public or private), the histopathological stage of the primary tumour (stages I-IIIA, DCIS), hormone status of tumour (presence of oestrogen and/or progesterone receptors), HER2 status, histological grade (1–3), lesion size, presence of lymphovascular invasion and the patient’s menopausal status. We used histopathological stage because while clinical staging contributes to initial surgeon decision making around breast conservation, histopathology results ultimately determine whether initial breast conservation needs to be converted to mastectomy, and the need for radiotherapy in many cases. Histopathology also provides more accurate information on tumour stage and patient prognosis [26]. The stages (I-IIIA) were classified based on the tumour size and lymph node involvement, referencing the American Society of Clinical Oncology Breast Cancer Staging [26]. This classification of stage is presented in supplementary Table 1.

The primary outcome variables were receipt of mastectomy, and receipt of radiotherapy for women who underwent BCS. The following were included as BCS: lumpectomy and any form of excision biopsy (where no subsequent excision was undertaken), wide local excision, partial mastectomy, and re-excision. The receipt of radiotherapy following BCS was recorded as a binary ‘yes’ or ‘no’ variable, as the data on courses of radiotherapy were unavailable from the NZBCR.

Those women who underwent BCS but did not receive adjuvant radiotherapy were categorised as referred or not referred for radiotherapy; each category was sub-grouped according to whether the clinician deemed not necessary, patient had declined, or patient was unfit for radiotherapy.

### Statistical analysis

Descriptive analyses were performed to present the numbers and percentages of the cohort in subgroups, and the distributions by ethnicity. Univariable analyses were used to observe the associations between the outcomes and individual variables of interest and reported using crude odds ratios (ORs) with 95% confidence intervals (95%CI). Multivariable logistic regression analyses were undertaken to identify the factors independently associated with each outcome. Separate analyses were conducted for the group of women with invasive disease and those with in situ disease including all the relevant demographic and clinicopathological factors for each group, without excluding the non-significant factors to minimise residual confounding. The effects were expressed as adjusted ORs with 95% CI, with p < 0.05 considered statistically significant. The analysis was undertaken using Python 3.7 statistical package.

### Ethics approval

The Te Rēhita Mate Ūtaetae, New Zealand Breast Cancer Registry, maintains its own governance and New Zealand Health and Disability Ethics Committee approval, using opt-out consent [3]. The analysis of the registry data for this study was additionally approved by the Health and Disability Ethics Committees (18/STH/165), and primary site localities at Waitematā (RM#13,920). All methods were carried out in accordance with relevant guidelines and regulations.

## Results

**Fig. 1:**
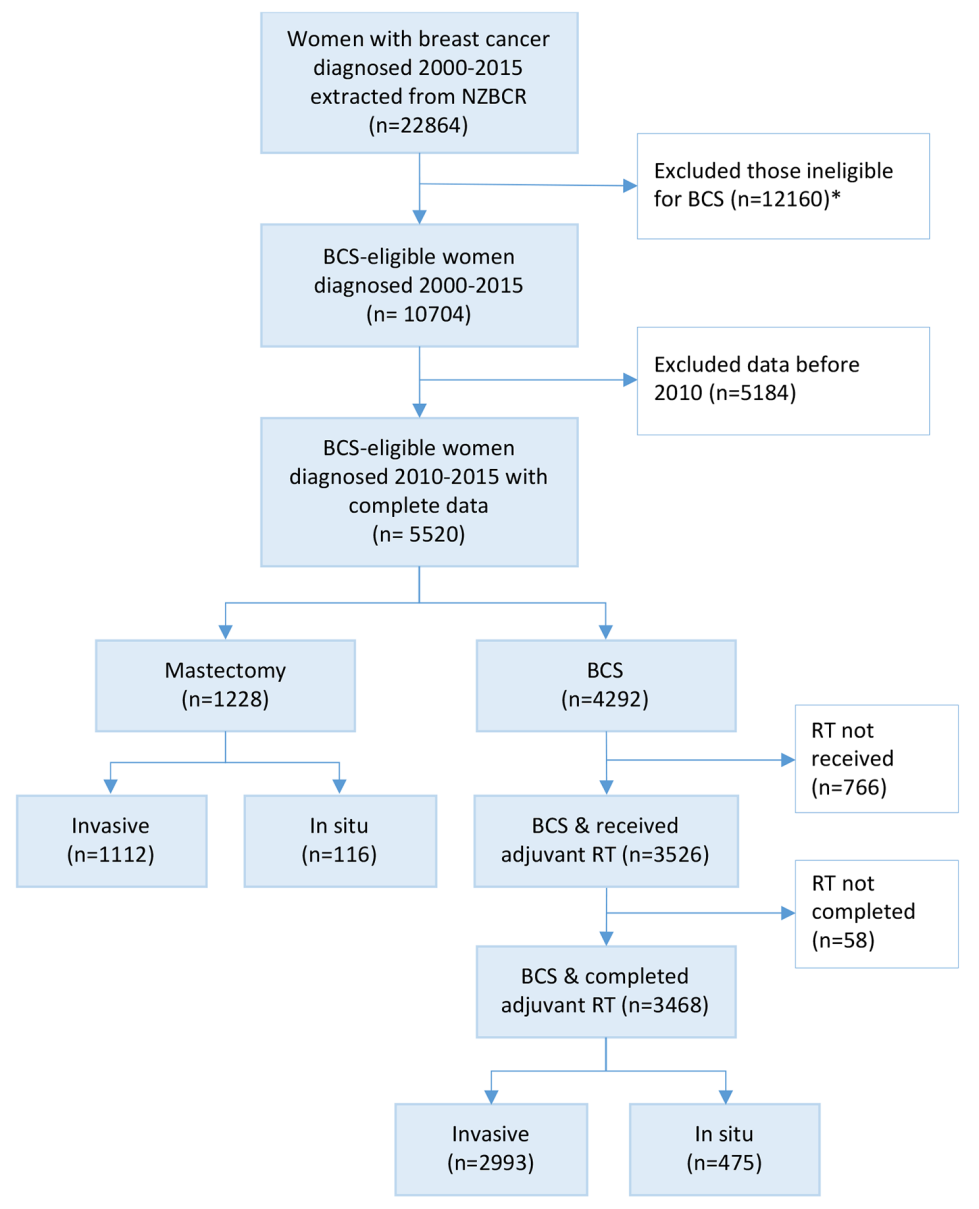
Flow diagram showing eligible patient selection. BCS = breast conserving surgery, RT = radiotherapy; * women with multiple lesions n=7021, men n=148, women with metastasis n=1770, pregnant women n=11, women with no surgery n=969, tumour size unknown or >30 mm n=2241

Of the total 22,864 registrations identified from the New Zealand Breast Cancer Registry (NZBCR) for 2000–2015, 10,704 women were eligible for analysis. After restricting the data to 2010–2015, the analyses included 5520 eligible women (Fig. 1). Most women (62.3%) were aged 50–69 years (Table 1). Māori women accounted for 8.2%, Pacific 4.0%, Asian 8.6% and Other ethnic group 78.7% of the cohort. Other population characteristics included invasive cancers (82.3%), a larger tumour size > 20 mm (44.4%), stage I (68.4%), lower grade 1–2 (62.2%), HER2 negative (82.8%) for those with invasive disease, and hormone receptor positive (84%) for those with invasive disease. Nearly two-third (63.7%) of women were screen-detected, and the majority were treated in the public health system.

**Table 1:** Patient characteristics, showing distributions by ethnicity

		Total	Ethnicity				
		N (%)	Māori	Pacific	Asian	Other	Unknown
Totals		5520	465 (8.2%)	223 (4.0%)	474 (8.6%)	4346 (78.7%)	12 (0.2%)
Age group							
	≤ 49 years	1192 (21.6%)	128 (27.5%)	58 (26.0%)	159 (33.5%)	847 (19.5%)	0 (0%)
	50–69 years	3440 (62.3%)	310 (66.7%)	152 (68.2%)	285 (60.1%)	2681 (61.7%)	12 (100%)
	70–79 years	636 (11.5%)	21 (4.5%)	11 (4.9%)	25 (5.3%)	579 (13.3%)	0 (0%)
	≥ 80 years	252 (4.6%)	6 (1.3%)	2 (0.9%)	5 (1.1%)	239 (5.5%)	0 (0%)
Cancer type							
	Invasive	4541 (82.3%)	398 (85.6%)	186 (83.4%)	343 (72.4%)	3607 (83.0%)	7 (82.3%)
	In situ	979 (17.7%)	67 (14.4%)	37 (16.6%)	131 (27.6%)	739 (17.0%)	5 (17.7%)
Size							
	< 10 mm	1558 (28.8%)	120 (25.8%)	58 (26%)	133 (28.1%)	1241 (28.6%)	6 (50%)
	11-19 mm	1478 (26.8%)	129 (27.7%)	72 (32.3%)	134 (28.3%)	1140 (26.2%)	3 (25%)
	> 20 mm	2484 (44.4%)	216 (46.5%)	93 (41.7%)	207 (43.7%)	1965 (45.2%)	3 (25%)
Stage							
	I	3778 (68.4%)	306 (65.8%)	139 (62.3%)	330 (69.6%)	2993 (68.9%)	10 (83.3%)
	IIA	1296 (23.5%)	116 (24.9%)	60 (26.9%)	113 (23.8%)	1005 (23.1%)	2 (16.7%)
	IIB	347 (6.3%)	36 (7.7%)	21 (9.4%)	25 (5.3%)	265 (6.1%)	0 (0%)
	IIIA	99 (1.8%)	7 (1.5%)	3 (1.3%)	6 (1.3%)	83 (1.9%)	0 (0%)
Grade							
	1	1346 (24.4%)	114 (24.5%)	45 (20.2%)	101 (21.3%)	1083 (24.9%)	3 (25%)
	2	2088 (37.8%)	197 (42.4%)	94 (42.2%)	152 (32.1%)	1642 (37.8%)	3 (25%)
	3	1057 (19.1%)	81 (17.4%)	44 (19.7%)	85 (17.9%)	846 (19.5%)	1 (8.3%)
	Unknown	1029 (18.7%)	73 (15.7%)	40 (17.9%)	136 (28.7%)	775 (17.8%)	5 (41.7%)
HER2 status (invasive)							
Subtotal		4541	398	186	343	3607	7
	Equivocal	137 (3.0%)	20 (5.0%)	4 (2.2%)	5 (1.5%)	107 (3.0%)	1 (14.3%)
	Negative	3759 (82.8%)	317 (79.6%)	154 (82.8%)	287 (83.7%)	2996 (83.1%)	5 (71.4%)
	Positive	438 (9.6%)	40 (10.1%)	21 (11.3%)	41 (12.0%)	335 (9.3%)	1 (14.3%)
	Unknown	207 (4.6%)	21 (5.3%)	7 (3.8%)	10 (2.9%)	169 (4.7%)	0 (0.0%)
HER2 status (in situ)							
Subtotal		979	67	37	131	739	5
	Equivocal	5 (0.5%)	0 (0.0%)	0 (0.0%)	0 (0.0%)	5 (0.7%)	0 (0.0%)
	Negative	50 (5.1%)	6 (9.0%)	1 (2.7%)	6 (4.6%)	36 (4.9%)	1 (20.0%)
	Positive	11 (1.1%)	1 (1.5%)	0 (0.0%)	1 (0.8%)	9 (1.2%)	0 (0.0%)
	Unknown	913 (93.3%)	60 (89.6%)	36 (97.3%)	124 (94.7%)	689 (93.2%)	4 (80.0%)
Hormone receptor (invasive)							
Subtotal		4541	398	186	343	3607	7
	Both neg	491 (10.8%)	18 (4.5%)	20 (10.8%)	38 (11.1%)	415 (11.5%)	0 (0.0%)
	Both pos	3025 (66.6%)	247 (62.1%)	141 (75.8%)	245 (71.4%)	2392 (66.3%)	0 (0.0%)
	Pos / neg	792 (17.4%)	88 (22.1%)	18 (9.7%)	57 (16.6%)	627 (17.4%)	2 (28.6%)
	Unknown	233 (5.1%)	45 (11.3%)	7 (3.8%)	3 (0.9%)	173 (4.8%)	5 (71.4%)
Hormone receptor (in situ)							
Subtotal		979	67	37	131	739	5
	Both neg	9 (0.9%)	0 (0.0%)	1 (2.7%)	1 (0.8%)	7 (0.9%)	0 (0.0%)
	Both pos	46 (4.7%)	4 (6.0%)	0 (0.0%)	5 (3.8%)	35 (4.7%)	2 (40.0%)
	Pos / neg	60 (6.1%)	5 (7.5%)	1 (2.7%)	6 (4.6%)	45 (6.1%)	3 (60.0%)
	Unknown	864 (88.3%)	58 (86.6%)	35 (94.6%)	119 (90.8%)	652 (88.2%)	0 (0.0%)
Mode of diagnosis							
	Screen-detected	3518 (63.7%)	322 (69.2%)	153 (68.6%)	299 (63.1%)	2738 (63%)	6 (50%)
	Symptomatic	2002 (36.3%)	143 (30.8%)	70 (31.4%)	175 (36.9%)	1608 (37%)	6 (50%)
Facility							
	Public	3292 (59.6%)	346 (74.4%%)	179 (80.3%)	313 (66.0%)	2447 (56.3%)	7 (58.3%)
	Private	1210 (21.9%)	28 (6.0%)	8 (3.6%)	111 (23.4%)	1058 (24.3%)	5 (41.7%)
	Unknown	1018 (18.4%)	91 (19.6%)	36 (16.1%)	50 (10.5%)	841 (19.4%)	0 (0%)

Regarding subgroups by ethnicity (Table 1), Asian women had a higher proportion of in situ cancers (27.6%) compared to women of other ethnicities (14–17%). Māori women were slightly more commonly diagnosed through screening (69.2%), followed by Pacific women (68.6%), compared to Asian and Other ethnicity (63%).

### Receipt of mastectomy in BCS eligible women

Of the 5520 BCS-eligible women, 1228 (22.2%) received mastectomy, with rates of 24.5% for women with invasive disease, and 11.8% for in situ disease (Table 2).

**Table 2:** Receipt of mastectomy in BCS-eligible women, by cancer type (invasive breast cancer and ductal carcinoma in situ)

	Category	Invasive						Ductal carcinoma in situ				
		Total	Mastectomy	Crude OR (95% CI)	Adjusted OR (95% CI)			Total	Mastectomy	Crude OR (95% CI)	Adjusted OR (95% CI)	
		N	n (%)					N	n (%)			
Total		4541	1112 (24.5)					979	116 (11.8)			
Age-group												
	≤ 49 years	949	235 (24.8)	1.46 (1.22–1.75)	0.96 (0.72–1.28)			243	30 (12.3)	1.38 (0.87–2.19)	0.86 (0.44–1.69)	
	50–69 years	2786	539 (19.3)	1	1			654	68 (10.4)	1	1	
	70–79 years	566	208 (36.7)	2.52 (2.05–2.52)	1.73 (1.37–2.18)	***		70	14 (20.0)	2.40 (1.26–4.56)	1.72 (0.80–3.70)	
	≥ 80 years	240	130 (54.2)	5.77 (4.34–7.67)	3.19 (2.31–4.42)	***		12	4 (33.3)	4.80 (1.40-16.39)	2.66 (0.65–10.82)	
Ethnicity												
	Māori	398	81 (20.4)	0.72 (0.55–0.95)	0.96 (0.72–1.29)			67	8 (11.9)	1.12 (0.52–2.42)	1.23 (0.53–2.86)	
	Pacific	186	51 (27.4)	1.22 (0.87–1.71)	1.12 (0.76–1.64)			37	4 (10.8)	1.00 (0.34–2.89)	0.64 (0.19–2.14)	
	Asian	343	134 (39.1)	2.18 (1.72–2.75)	2.20 (1.7–2.86)	***		131	18 (13.7)	1.31 (0.76–2.27)	1.31 (0.73–2.38)	
	Other	3607	844 (23.4)	1	1			739	84 (11.4)	1	1	
	Unknown	7	2 (28.6)	-	-			5	2 (40.0)	-	-	
Mode of Diagnosis												
	Screen-detected	2692	434 (16.1)	1	1			826	78 (9.4)	1	1	
	Symptomatic	1849	678 (36.7)	3.00 (2.60–3.47)	1.97 (1.66–2.35)	***		153	38 (24.8)	3.07 (1.96–4.80)	2.55 (1.48–4.41)	***
Year of Diagnosis												
	Year 2010/11	1425	371 (26.0)	1.18 (0.99–1.39)	1.24 (1.03–1.49)	*		310	41 (13.2)	1.23 (0.78–1.96)	1.06 (0.64–1.74)	
	Year 2012/13	1458	356 (24.4)	1.04 (0.8 8-1.24)	1.15 (0.96–1.38)			281	31 (11.0)	0.97 (0.59–1.60)	0.87 (0.50–1.5)	
	Year 2014/15	1658	385 (23.2)	1	1			388	44 (11.3)	1	1	
Menopausal Status												
	Pre-menopausal	1133	278 (24.5)	1.30 (0.91–1.88)	0.99 (0.67–1.46)			315	43 (13.7)	1.47 (0.50–4.33)	1.26 (0.38–4.17)	
	Peri-menopausal	231	46 (19.9)	1	1			44	5 (11.4)	1	1	
	Post-menopausal	3177	788 (24.8)	1.34 (0.94–1.89)	0.97 (0.66–1.41)			620	68 (11.0)	1.13 (0.39–3.28)	0.91 (0.28–2.89)	
Facility Type												
	Private	983	225 (22.9)	1.14 (0.90–1.29)	0.98 (0.78–1.23)			227	26 (11.5)	1.14 (0.70–1.85)	1.13 (0.6–2.14)	
	Public	2704	671 (24.8)	1	1			588	65 (11.1)	1	1	
	Unknown	854	216 (25.3)	-	-			164	25 (15.2)	-	-	
Stage												
	I	2799	492 (17.6)	1	1							
	IIA	1296	428 (33.0)	2.07 (1.77–2.44)	1.57 (1.22–2.01)	**						
	IIB	347	142 (40.9)	3.45 (2.64–4.50)	1.99 (1.37–2.91)	***						
	IIIA	99	50 (50.5)	3.84 (2.49–5.94)	3.72 (2.31–6.01)	***						
HER2 Status												
	Equivocal	123	20 (16.3)	0.65 (0.39–1.08)	0.84 (0.48–1.45)							
	Negative	3744	828 (22.1)	1	1							
	Positive	433	145 (33.5)	1.78 (1.42–2.21)	1.55 (1.21–1.97)	***						
	Unknown	241	119 (49.4)	-	-							
Grade												
	1	1346	224 (16.6)	1	1							
	2	2088	527 (25.2)	1.71 (1.42–2.04)	1.34 (1.1–1.63)	**						
	3	1055	341 (32.3)	2.33 (1.90–2.85)	1.28 (0.99–1.64)							
	Unknown	52	20 (38.5)	-	-							
Size												
	< 10 mm	1149	178 (15.5)	1	1							
	11-19 mm	1265	488 (38.6)	3.82 (3.12–4.67)	1.74 (1.24–2.44)	**						
	> 20 mm	2127	446 (21.0)	1.81 (1.51–2.17)	1.29 (1.04–1.60)	***						
Lymphovascular Invasion												
	No	3720	869 (23.4)	1	1							
	Yes	792	235 (29.7)	1.34 (1.13–1.61)	0.84 (0.69–1.03)	*						
	Unknown	29	8 (27.6)	-	-							
Hormone receptor												
	Both negative	491	179 (36.5)	1.87 (1.51–2.31)	1.5 (1.16–1.93)	***						
	Both positive	3025	725 (24.0)	1	1							
	One pos/one neg	792	152 (19.2)	0.72 (0.59–0.89)	1.10 (0.87–1.39)							
	Unknown	233	56 (24.0)	-	-							
Rurality												
	Rural	187	30 (16.0)	0.64 (0.44–0.93)	1.03 (0.68–1.55)							
	Urban	2606	630 (24.2)	1	1							
	Unknown	1748	452 (25.9)	-	-							
Deprivation Status												
	NZDep Quintile 1	621	140 (22.5)	1	1							
	NZDep Quintile 2	745	178 (23.9)	1.15 (0.88–1.49)	1.00 (0.76–1.32)							
	NZDep Quintile 3	643	148 (23.0)	1.05 (0.80–1.38)	1.16 (0.86–1.56)							
	NZDep Quintile 4	558	133 (23.8)	1.05 (0.79–1.4)	1.22 (0.89–1.68)							
	NZDep Quintile 5	142	35 (24.6)	1.17 (0.75–1.81)	0.98 (0.59–1.64)							
	Unknown	1832	478 (26.1)	-	-							

*p ≤ 0.05; **p ≤ 0.01; ***p ≤ 0.001; OR = odds ratios based on logistic regression, unknown groups were omitted; 95% CI = 95% confidence interval, those showing significant are underlined. *Note*: In ductal carcinoma in situ analysis, stage, grade, size, HER2 status, hormone receptor status, lymphovascular invasion, rurality and deprivation were omitted

**Fig. 2:**
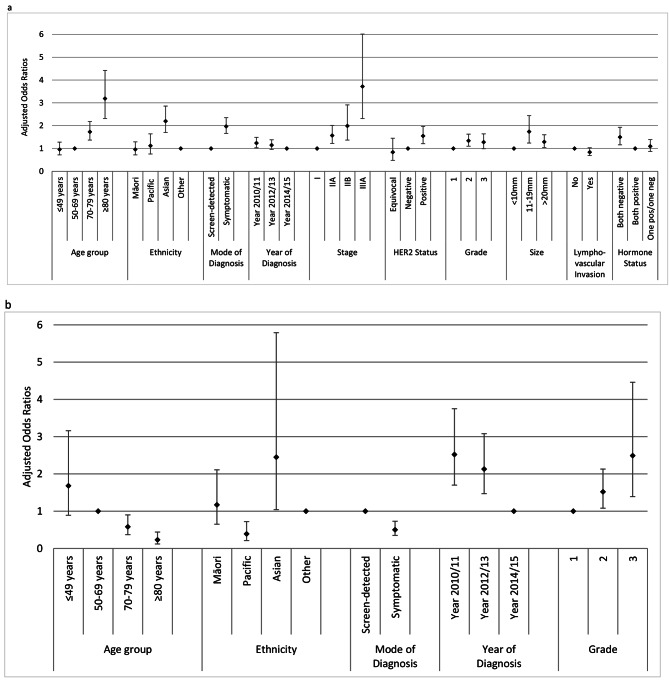
Factors significantly affecting **a**) receipt of mastectomy in women with invasive breast cancer who were eligible for breast conserving surgery (BCS); **b**) receipt of adjuvant radiotherapy following BCS in women with invasive breast cancer. Note: Based on the multivariable logistic regression model; only significant factors are shown; factors not shown in figure (**a**) are menopausal status, facility type, rurality, and deprivation status; and factors not shown in figure (**b**) are menopausal status, facility type, stage, HER2 status, size, lymphovascular invasion, hormone status, rurality, and deprivation status

For the invasive group, the univariable analysis indicated that the receipt of mastectomy was about two times higher in Asian women (OR 2.18; 95% CI 1.72–2.75), with no significant differences for Māori and Pacific women when compared to women of Other ethnicity. The multivariable analysis showed similar ethnic differences. The other factors significantly associated with higher mastectomy rate included age ≥ 70 years, symptomatic disease, more advanced stages (II and III), grade 2, larger size (≥ 11 mm), hormone receptor-negative, HER2-positive, and diagnosed in 2010/11 (Table 2; Fig. 2a). No difference was seen by rurality.

For the in situ group, both univariable and multivariable analyses showed no significant differences in receiving mastectomy by ethnicity or other factors, except that mastectomy was more likely to be received in women with symptomatic disease than those with screen-detected disease (OR 2.55; 95% CI 1.48–4.41) (Table 2).

### Receipt of adjuvant radiotherapy following BCS

Of the 3809 women who received BCS, 3468 (91%) received adjuvant radiotherapy, with rates of 93.5% for women with invasive disease, and 78.3% for in situ disease (Table 3).

**Table 3:** Receipt of adjuvant radiotherapy in women who underwent BCS as final surgery, by cancer type (invasive breast cancer and ductal carcinoma in situ)

		Invasive						Ductal carcinoma in situ				
	Category	Total	Adjuvant radiotherapy	Crude OR (95% CI)	Adjusted OR (95% CI)			Total	Adjuvant radiotherapy	Crude OR (95% CI)	Adjusted OR (95% CI)	
		N	n (%)					N	n (%)			
Total		3202	2993 (93.5)					607	475 (78.3)			
Age-group												
	≤ 49 years	666	640 (96.1)	1.48 (0.96–2.28)	1.68 (0.89–3.16)			149	112 (75.2)	0.73 (0.47–1.15)	0.46 (0.22–0.96)	*
	50–69 years	2121	1999 (94.2)	1	1			423	343 (81.1)	1	1	
	70–79 years	332	294 (88.6)	0.47 (0.32–0.69)	0.58 (0.37–0.90)	*		32	21 (65.6)	0.45 (0.21–0.97)	0.47 (0.20–1.11)	
	≥ 80 years	83	60 (72.3)	0.14 (0.08–0.23)	0.23 (0.12–0.44)	***		3	1 (33.3)	-	-	
Ethnicity												
	Māori	299	284 (95.0)	1.26 (0.74–2.13)	1.17 (0.65–2.11)			38	33 (86.8)	1.75 (0.67–4.60)	1.59 (0.54–4.65)	
	Pacific	128	111 (86.7)	0.47 (0.27–0.80)	0.39 (0.21–0.72)	**		21	15 (71.4)	0.66 (0.25–1.75)	0.31 (0.10–0.92)	*
	Asian	195	188 (96.4)	2.25 (0.98–5.14)	2.45 (1.04–5.79)			77	54 (70.1)	0.76 (0.43–1.32)	0.56 (0.31–1.05)	
	Other	2575	2406 (93.4)	1	1			471	373 (79.2)	1	1	
	Unknown	5	4 (80.0)	-	-			-	-	-	-	
Mode of Diagnosis												
	Screen-detected	2128	2016 (94.7)	1	1			544	429 (78.9)	1	1	
	Symptomatic	1074	977 (91.0)	0.58 (0.44–0.77)	0.5 (0.35–0.730)	***		63	46 (73.0)	0.76 (0.41–1.39)	0.97 (0.48–1.95)	
Year of Diagnosis												
	Year 2010/11	985	946 (96.0)	2.50 (1.73–3.62)	2.52 (1.70–3.75)	***		182	152 (83.5)	1.73 (1.05–2.84)	1.66 (0.97–2.83)	
	Year 2012/13	1022	969 (94.8)	2.03 (1.44–2.85)	2.13 (1.47–3.08)	***		175	135 (77.1)	0.99 (0.63–1.56)	0.99 (0.6–1.62)	
	Year 2014/15	1195	1078 (90.2)	1	1			250	188 (75.2)	1	1	
Menopausal Status												
	Pre-menopausal	794	757 (95.3)	0.98 (0.45–2.14)	0.82 (0.35–1.92)			187	148 (79.1)	1.51 (0.62–3.66)	1.94 (0.72–5.28)	
	Peri-menopausal	175	167 (95.4)	1	1			30	21 (70.0)	1	1	
	Post-menopausal	2233	2069 (92.7)	0.61 (0.29–1.26)	0.97 (0.45–2.11)			390	306 (78.5)	1.40 (0.60–3.28)	1.12 (0.43–2.89)	
Facility Type												
	Private	710	678 (95.5)	2.06 (1.39–3.05)	1.51 (0.94–2.44)			147	110 (74.8)	0.66 (0.43–1.03)	0.67 (0.38–1.17)	
	Public	1925	1763 (91.6)	1	1			391	313 (80.1)	1	1	
	Unknown	567	552 (97.4)	-	-			69	52 (75.4)	0.79 (0.43–1.45)	0.63 (0.08–5.25)	
Stage												
	I	2153	2001 (92.9)	1	1							
	IIA	812	771 (95.0)	1.83 (1.22–2.74)	1.55 (0.88–2.71)							
	IIB	189	176 (93.1)	0.99 (0.51–1.92)	0.85 (0.29–2.48)							
	IIIA	48	45 (93.8)	1.12 (0.34–3.65)	0.90 (0.24–3.43)							
HER2 Status												
	Equivocal	100	94 (94.0)	1.09 (0.47–2.53)	1.20 (0.41–2.47)							
	Negative	2729	2554 (93.6)	1	1							
	Positive	269	258 (95.9)	1.78 (0.93–3.42)	1.19 (0.6–2.39)							
	Unknown	104	87 (83.7)	-	-							
Grade												
	1	1053	966 (91.7)	1	1							
	2	1454	1361 (93.6)	1.30 (0.96–1.76)	1.52 (1.08–2.13)	**						
	3	672	648 (96.4)	2.34 (1.47–3.72)	2.49 (1.39–4.46)	***						
	Unknown	23	18 (78.3)	-	-							
Size												
	< 10 mm	884	814 (92.1)	1	1							
	11-19 mm	1601	1504 (93.9)	1.16 (0.86–1.57)	1.27 (0.9–1.79)							
	> 20 mm	717	675 (94.1)	1.64 (1.03–2.61)	1.76 (0.78–3.98)							
Lymphovascular Invasion												
	No	2650	2471 (93.2)	1	1							
	Yes	532	503 (94.5)	1.22 (0.82–1.83)	0.95 (0.59–1.51)							
	Unknown	20	19 (95.0)	-	-							
Hormone receptor												
	Both negative	290	279 (96.2)	1.43 (0.78–2.61)	0.87 (0.43–1.75)							
	Both positive	2141	2006 (93.7)	1	1							
	One pos/one neg	609	564 (92.6)	0.81 (0.57–1.14)	0.93 (0.59–1.47)							
	Unknown	162	144 (88.9)	-	-							
Rurality												
	Rural	151	139 (92.1)	0.8 (0.44–1.47)	0.76 (0.38–1.49)							
	Urban	1843	1712 (92.9)	1	1							
	Unknown	1208	1142 (94.5)	-	-							
Deprivation Status												
	NZDep Quintile 1	440	411 (93.4)	1	1							
	NZDep Quintile 2	529	493 (93.2)	1.04 (0.63–1.74)	1.03 (0.6–1.76)							
	NZDep Quintile 3	471	439 (93.2)	0.99 (0.59–1.65)	1.05 (0.6–1.84)							
	NZDep Quintile 4	396	357 (90.2)	0.65 (0.40–1.07)	0.61 (0.34–1.09)							
	NZDep Quintile 5	102	97 (95.1)	1.45 (0.55–3.83)	1.17 (0.39–3.49)							
	Unknown	1264	1196 (94.6)	-	-							

*p ≤ 0.05; **p ≤ 0.01; ***p ≤ 0.001; OR = odds ratios based on logistic regression, unknown groups were omitted; 95% CI = 95% confidence interval, those showing significant are underlined. *Note*: In ductal carcinoma in situ analysis, stage, grade, size, HER2 status, hormone receptor status, lymphovascular invasion, rurality and deprivation were omitted

For the invasive group, the univariable analysis showed that the receipt of adjuvant radiotherapy was significantly less likely in Pacific women (OR 0.47; 95% CI 0.27–0.80), with no significant differences for Māori and Asian, compared to Other ethnicity. When adjusted for other factors, the association with Pacific ethnicity showed a larger effect (OR 0.39; 95% CI 0.21–0.72), and the association with Asian (more likely) was significant (OR 2.45; 95% CI 1.04–5.79). Adjuvant radiotherapy was also less likely to be received in women aged ≥ 70 years, and in symptomatic disease. In contrast, the receipt of adjuvant radiotherapy was higher in registrations before 2014, and those having higher grade tumours (Table 3; Fig. 2b).

For the in situ group, similarly, the receipt of adjuvant radiotherapy was significantly less likely in Pacific women, showing a larger effect in multivariable analysis: OR 0.31 (95% CI 0.10–0.92), with no significant associations with Māori and Asian, compared to Other ethnicity. Adjuvant radiotherapy was also less likely to be received in younger women of ≤ 49 years than older women (Table 3).

Regarding referral status among women who underwent BCS without adjuvant radiotherapy, ‘not referred - deemed not necessary’ category was the most common (8%), followed by ‘referred - patient declined’ (5%) (Table 4). In the category ‘not referred - deemed not necessary’, Asian women contributed the highest percentage (13%) compared to other ethnic groups (7–9%); and so did the women aged ≥ 80 years (23%) compared to other age groups (8–9%). In the category ‘referred - patient declined’, Pacific women contributed the highest proportion (10%) compared to other ethnic groups (3–7%); and so did the women aged ≥ 80 years (14%) compared to other age groups (4–6%).

**Table 4:** Radiotherapy referral data for women who underwent BCS, by ethnicity and age group

		Radiotherapy received	Radiotherapy not received							Unknown	Total
			Not referred -			Not yet	Referred -				
			Deemed not necessary	Patient declined	Patient unfit		Deemed not necessary	Patient declined	Patient unfit		
Total		3526 (82%)	361 (8%)	1 (0%)	2 (0%)	109 (3%)	89 (2%)	196 (5%)	0 (0%)	5(0%)	4292
Ethnicity											
	Māori	317 (84%)	28 (7%)	0 (0%)	0 (0%)	11 (3%)	8 (2%)	11 (3%)	1 (0%)	0 (0%)	376
	Pacific	126 (75%)	15 (9%)	0 (0%)	0 (0%)	3 (2%)	6 (4%)	17 (10%)	0 (0%)	1 (1%)	168
	Asian	246 (76%)	42 (13%)	0 (0%)	0 (0%)	8 (2%)	5 (2%)	21 (7%)	0 (0%)	0 (0%)	322
	Others	2831 (83%)	276 (8%)	1 (0%)	2 (0%)	87 (3%)	68 (2%)	147 (4%)	0 (0%)	4 (0%)	3418
	Unknown	6 (75%)	0 (0%)	0 (0%)	0 (0%)	0 (0%)	2 (25%)	0 (0%)	1 (13%)	0 (0%)	8
Age group											
	≤ 49 years	758 (82%)	80 (9%)	0 (0%)	2 (0%)	31 (3%)	15 (2%)	40 (4%)	0 (0%)	1 (0%)	927
	50–69 years	2386 (84%)	222 (8%)	0 (0%)	0 (0%)	60 (2%)	50 (2%)	113 (4%)	1 (0%)	1 (0%)	2833
	70–79 years	319 (77%)	32 (8%)	1 (0%)	0 (0%)	14 (3%)	18 (4%)	26 (6%)	1 (0%)	3 (1%)	414
	≥ 80 years	63 (53%)	27 (23%)	0 (0%)	0 (0%)	4 (3%)	6 (5%)	17 (14%)	0 (0%)	1 (1%)	118

## Discussion

Our research, for the first time, has examined the treatment decision for mastectomy and adjuvant radiotherapy after breast conserving surgery (BCS) in the selected cohort of New Zealand women who were eligible for BCS, focusing on ethnic differences. We assessed 5520 eligible women, including 4541 invasive and 979 in situ patients over a six-year period.

### Receipt of mastectomy in BCS eligible women

We found that 22.2% of all BCS-eligible women in our study received mastectomy during 2010–2015, with a higher proportion in women with invasive disease (24.5%) and a much lower proportion in those with in situ disease (11.8%). These mastectomy rates appear lower than a US national study of 1.2 million BCS-eligible women, which reported a mastectomy rate of 35.5% overall – 37.9% invasive and 19.3% in situ [27]. A 2009–2014 Australian study of 24,666 breast cancer patients who underwent breast surgery also reported a higher mastectomy rate of 36%, although eligibility for BCS was unknown [28].

Our study showed that Asian women were twice as likely to receive a mastectomy for invasive disease compared to women of Other ethnicity (p < 0.001), but no significant difference was seen for Māori and Pacific women. The finding of the higher mastectomy rates for Asian is supported by a previous New Zealand study [29]. While the specific underlying reasons for this are unknown, the potentially related factors may include smaller average breast size in relation to tumour size, cultural views, and younger age at diagnosis in Asian women with breast cancer, compared to New Zealand European women [29]. A previous study involving women with tumour size up to 50 mm evidenced that Māori were significantly more likely to receive mastectomy overall than Other ethnicity (OR 1.45, 95% CI 1.07–1.95) [15]. Māori and Pacific women were also frequently diagnosed via the symptomatic pathway, resulting in more advanced disease and more aggressive treatment in these groups [5, 15]. In our study using a BCS restricted cohort, surprisingly, the proportion of screen-detected cancers was higher for Māori and Pacific women than that of Asian and Other ethnicity; and screen-detected cancers were associated with decreased likelihood of mastectomy. Our findings that receipt of mastectomy for Māori and Pacific women was similar to that seen for Other ethnicities may result from the exclusion of larger or multifocal tumours from the study sample. We note that breast density and breast volume, on which the data were unavailable in our study, may be relevant to the choice of surgery in different ethnic groups.

Our research indicated a higher rate of mastectomy in women of older age, symptomatic disease, higher stage, hormone receptor-negative, and HER2-positive, in accordance with previous research [15, 30, 31]. An earlier study in the Auckland and Waikato regions [16] found that receipt of mastectomy also varied by socioeconomic status and facility type, for which, our study showed no significant variation. Our finding of non-variation for rurality may partly be explained by the coverage of the NZBCR, which at the point of data extraction has only just consolidated the Auckland, Waikato, Wellington and Christchurch datasets, while including other cancer centres (e.g., Southern and Midcentral regions) may provide a better comparison for rural populations.

Our study observed a decrease in receipt of mastectomy over the 6-year period. This reflects a change in practice and international trend of increasing BCS [32–34], research evidence and growing experience with oncoplastic breast techniques that allow resection of larger breast volumes while retaining good aesthetic outcomes. The change may also be related to greater use of neoadjuvant therapy which can reduce tumour size and permit consideration of BCS as a surgical option in a larger number of patients.

### Receipt of adjuvant radiotherapy following BCS

Our study found that most women (91.1%) who had BCS received adjuvant radiotherapy, with a higher rate for invasive disease (93.5%) than for in situ disease (78.8%). The rate of adjuvant radiotherapy after BCS varies widely across different studies and populations; for example, it was 66.2% in a US breast cancer cohort [18], and 81% in an Australian breast cancer cohort [28]. These international studies suggested the lower uptake of adjuvant radiotherapy in certain patient groups may be related to older age, comorbidities, lower socioeconomic position, and distance to radiotherapy centre.

Our analysis revealed that adjuvant radiotherapy was much lower in Pacific women and higher in Asian women with invasive disease than those of Other ethnicity, showing no significant difference for Māori, after adjusting for other demographic and clinicopathological factors. Our research also observed that a higher proportion of Pacific women declined radiotherapy after being referred compared to women of the reference Other ethnic group. Potential reasons for the lower rate of radiotherapy in Pacific women include health literacy, cultural views, socioeconomic inequities and health systems factors such as lack of continuity, difficulty in access and critical shortage in Pacific healthcare workforce [35]; whereas potential reasons for the higher rate of adjuvant radiotherapy in Asian women include better treatment compliance among others [29]. Previous studies found ethnic differences in receipt of radiotherapy for both Māori and Pacific women, and were found to be contributory to overall survival differences [5], whereas our study did not demonstrate a disparity for Māori women in the subset of BCS-eligible women. Further research will be beneficial to explain the variations in rates of adjuvant radiotherapy among different ethnic groups.

Age contributed the largest significant determinant to receiving adjuvant radiotherapy after BCS – older women ≥ 70 years were substantially less likely to receive radiotherapy compared to younger women. The referral data indicated that women ≥ 80 years were more likely to not be referred as the clinician deemed it not necessary, and to decline treatment after referral than women of younger age groups. How the variable ‘deemed not necessary’ is determined and collected (its completeness or representativeness of clinical decision making) is not clear from the dataset. Evidence suggests there are various factors that lead to age differences in receiving radiotherapy, including evidence on lower treatment benefit for older women [36–38], and studies which discuss how information on treatment options is presented, the impact of age on health literacy [39–42], patient’s and/or clinician’s views or preference related to a type of surgery (or consequent need for radiotherapy or multiple operations), as well as the presence of comorbidities in older women [43]. Presence of comorbidities and less treatment benefit amongst older women may also be related to the lower referral rate for adjuvant radiotherapy.

We found, unexpectedly, that symptomatic women were less likely to receive radiotherapy than those that were screen-detected, particularly as a symptomatic woman may have a higher grade or more advanced stage cancer compared to a screen detected cancer, and therefore optimising local control through adjuvant radiotherapy would be important. On further investigation, this finding appears to reflect the gap reported in the 2016 New Zealand audit, where 93.3% of screen-detected women were referred for radiotherapy compared to 86.9% of symptomatic women [43]. International analyses have reported a range of factors associated with non-receipt of radiotherapy including socioeconomic status, ethnicity, rurality, and distance from a treatment centre [44–46].

Our study showed a downward trend in rates of receiving adjuvant radiotherapy over 2010–2015, from 96 to 90%. A 2011 US study reported a fluctuating range of 61–70% among early stage breast cancer patients over the past decade [18]. It is expected to see ongoing changes in receipt of adjuvant radiotherapy rates with time as radiotherapy guidelines are continually evolving for breast cancer and evidence from clinical trials enters routine clinical practice [47]. The START trial [12] in 2013 and the FASTFORWARD study [48] in 2020 demonstrated that reduced duration of radiotherapy (3 weeks and 1 week, respectively) by increasing the dose per daily treatment could retain comparable cancer control and cosmetic outcomes in selected patient groups. The EXPERT trial [49], which started recruitment in New Zealand in 2017, is assessing the impact of omitting adjuvant radiotherapy in low risk breast cancers (luminal A and low recurrence score on PAM50 testing) after BCS. The downward trend of adjuvant radiotherapy use likely to represent changes in clinical practice, which may be related to updates in clinical trial-based evidence.

### Strengths and limitations

Our research was population-based and included a large cohort of women with breast cancer, covering two-thirds of the whole breast cancer population in New Zealand. Our study’s focus on BCS-eligible women facilitated clear interpretation of effect of ethnicity by excluding women with more complex clinical situations, and ethnic differences driven by late stage at presentation. Although a clinically determined variable of ‘BCS-eligible’ was not available, we used as much relevant information as possible in the analysis; however, some information that may be relevant in this study was unavailable, such as breast density, breast volume, tumour size in relation to breast volume, tumour location, family history/genetic status, patient comorbidities, and contraindications for surgery or adjuvant radiotherapy following BCS. The external validity of this study may be limited given the regional differences in the distribution of ethnicity, age and deprivation score.

## Conclusion

In New Zealand women with early breast cancer eligible for BCS, the mastectomy rates were similar in Māori and Pacific women, but significantly higher in Asian women with invasive disease, compared to the Other ethnic group, after adjusting for demographic and clinicopathological factors. Rates of radiotherapy use are high by international standards. The adjuvant radiotherapy rates after BCS were similar in Māori, but substantially lower in Pacific women and higher in Asian women with invasive disease, compared to the Other ethnic group, and lower for symptomatic women. Both of these findings are of concern. Other significant factors affecting treatment choices included age, diagnosis year, mode of detection, and tumour factors such as grade. The radiotherapy referrals revealed a higher rate of non-referral due to it being deemed unnecessary in Asian women, and a higher rate of self-decline after being referred in Pacific women, compared to Other ethnic groups. Further research is required to understand differences by ethnicity in both rates of mastectomy and lower rates of radiotherapy after BCS for Pacific women, and the reasons for non-referral by clinicians.

### Electronic supplementary material

Below is the link to the electronic supplementary material.

Supplementary Material 1

## Data Availability

The data used and analysed during the current study contain identifiable individual patient information. The data are not publicly available due to the data confidentiality and privacy restrictions but are available from the corresponding author on reasonable request and corresponding approvals. Please contact Health and Disability Ethics Committees at hdecs@health.govt.nz for ethics queries.

## List of abbreviations

BCS: Breast Conserving Surgery
DCIS: Ductal Carcinoma In Situ
NZBCF: New Zealand Breast Cancer Foundation
NZBCR: New Zealand Breast Cancer Register
NHI: National Health Index
MELAA: Middle Eastern Latin American and African
