# Supplementary material for: Receipt of mastectomy and adjuvant radiotherapy following breast conserving surgery (BCS) in New Zealand women with BCS-eligible breast cancer, 2010–2015: an observational study focusing on ethnic differences
Source: BMC Cancer. 2023 Aug 17;23:766. doi: 10.1186/s12885-023-11248-9 (PMC10436661; doi:10.1186/s12885-023-11248-9)
Supplement: Supplementary file 1 — Supplementary Material 1 [file 12885_2023_11248_MOESM1_ESM.docx]

Supplementary Table 1. Cancer stage classification

| Tumour Size | N Node | TNMT | Size from TNM | Stage |
| --- | --- | --- | --- | --- |
| 0-19mm | 0 |  |  | IA |
| 0-19mm |  | PN1mi |  | IB |
| 0-19mm |  | PT1mip |  | IB |
| 0-19mm |  | PT1misn |  | IB |
| 0-19mm | 1-3 |  |  | IIA |
| 20-50mm | 0 |  |  | IIA |
| 20-50mm | 1-3 |  |  | IIB |
| >50mm | 0 |  |  | IIB |
| 0-19mm | 4-9 |  |  | IIIA |
| 20-50mm | 4-9 |  |  | IIIA |
| >50mm | 1-3 |  |  | IIIA |
| 0-19mm | S0 |  |  | IA |
| 0-19mm | S1-3 |  |  | IIA |
| 20-50mm | S0 |  |  | IIA |
| 20-50mm | S1-3 |  |  | IIB |
| >50mm | S0 |  |  | IIB |
| 0-19mm | S4-9 |  |  | IIIA |
| 20-50mm | S4-9 |  |  | IIIA |
| >50mm | S1-3 |  |  | IIIA |
|  |  | T0 | No cancer |  |
|  |  | T1mi | ≤1 mm |  |
|  |  | T1a | >1 to 5 mm |  |
|  |  | T1b | >5 to 10 mm |  |
|  |  | T1c | >10 to 20 mm |  |
|  |  | T2 | >20 to 50 mm |  |
|  |  | T3 | >50 mm |  |

Reference: American Society of Clinical Oncology. Breast Cancer: Stages. CancerNet 2017. https://www.cancer.net/cancer-types/breast-cancer/stages (accessed June 29, 2023).
